# Nodular Epithelial Hyperplasia after Photorefractive Keratectomy Followed by Corneal Collagen Cross-Linking

**DOI:** 10.1155/2013/953267

**Published:** 2013-04-04

**Authors:** Ayla Bogoni, Liberdade Cezaro Salerno, Vinícius Coral Ghanem, Ramon Coral Ghanem

**Affiliations:** ^1^Sadalla Amin Ghanem Eye Hospital, Rua Camboriú, 35, 89216-222 Joinville, SC, Brazil; ^2^University of São Paulo, São Paulo, Brazil

## Abstract

This study describes a case of nodular epithelial hyperplasia and stromal alterations in a patient with keratoconus who was submitted to topography-guided photorefractive keratectomy (PRK) followed by corneal collagen cross-linking. Debridement of the epithelial nodule was performed. After a 2-year followup, a new topography-guided PRK was indicated.

## 1. Introduction

Keratoconus is a progressive and bilateral noninflammatory corneal disorder characterized by localized protrusion of the cornea with stromal thinning [1, 2]. Corneal topography suggesting keratoconus is associated with greater risk of ectasia after refractive surgery [3], although some authors report good results in surface refractive surgery for eyes with keratoconus or suspected keratoconus [1, 4].

Treatment using corneal collagen cross-linking (CXL) with riboflavin and ultraviolet-A (UVA) light aims to stabilize the progression of keratoconus and other corneal ectasia by inducing cross-links between the stromal collagen molecules, thus promoting greater corneal stromal biomechanical stability [5]. Combined with CXL, refractive surgery becomes a feasible option for selected cases of keratoconus [1].

We report a case in which a patient with keratoconus who was submitted to the photorefractive keratectomy (PRK) technique followed by CXL subsequently presented a central nodular epithelial hyperplasia. This has not been previously described in the research literature.

## 2. Case Report

**Figure 1:**
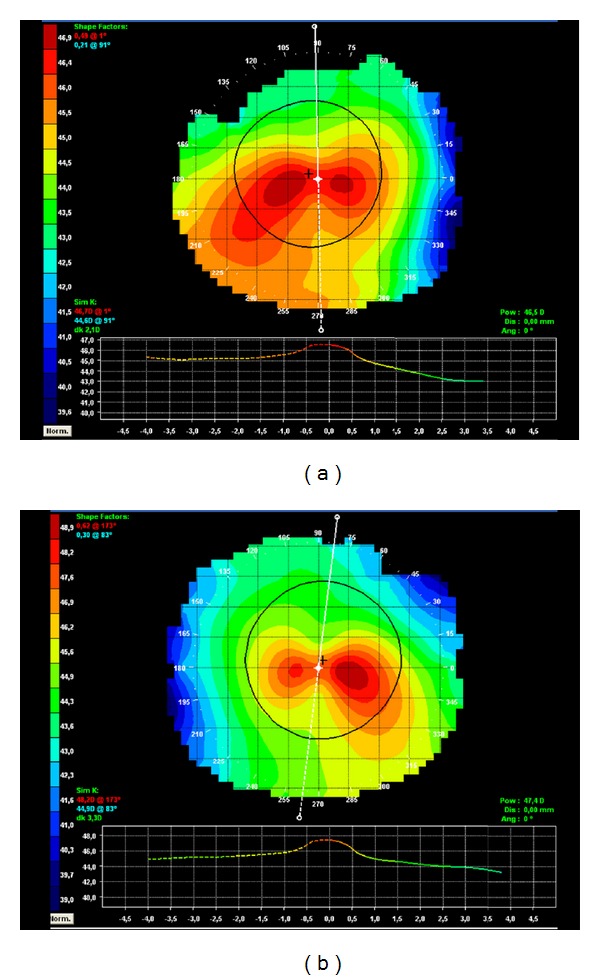
Preoperative corneal topography: (a) RE, (b) LE.

**Figure 2:**
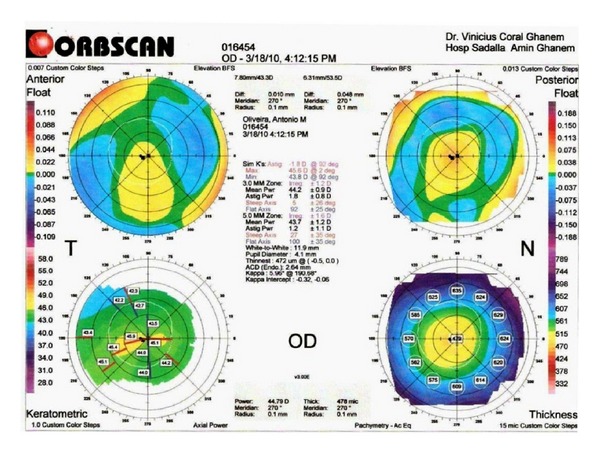
RE preoperative pachymetry map generated by Orbscan II, with thinnest corneal point of 472 *μ*m.

A 40-year-old Caucasian man with keratoconus was evaluated for refractive surgery in March 2010. Visual acuity (VA) in the right eye (RE) with correction was 20/25 (+1.25, −2.25, 105°) and in the left eye (LE) was 20/30 (+0.50, −3.00, 85°). The topography showed keratoconus with maximum apical keratometry of 46.9 D in the RE and 48.9 D in the LE (Figure 1). The thinnest point on the pachymetry map (Orbscan II) was 472 *μ*m in the RE (Figure 2) and 487 *μ*m in the LE.

Considering that very few studies have investigated the results of combining PRK with CXL, we decided to initially perform the procedure on the nondominant eye only (RE).

Topical anesthesia was performed for PRK with 2 drops of proximetacaine hydrochloride 0.5% (Anestalcon, Alcon, São Paulo, Brazil) 15 minutes before surgery, with a 5-minute interval between drops, and 1 drop of tetracaine hydrochloride 1% and phenylephrine hydrochloride 0.1% (Anestésico, Allergan, São Paulo, Brazil) moments before the procedure. Using a slit lamp, the horizontal meridian (0° and 180°) was marked with a gentian violet pen while the patient was in the surgical anteroom. In order to compensate for cyclotorsion, these markings were adjusted to the horizontal line of the microscope eyepiece. Hemifacial asepsis was performed with povidone 10% without eye surface contact.

**Figure 3:**
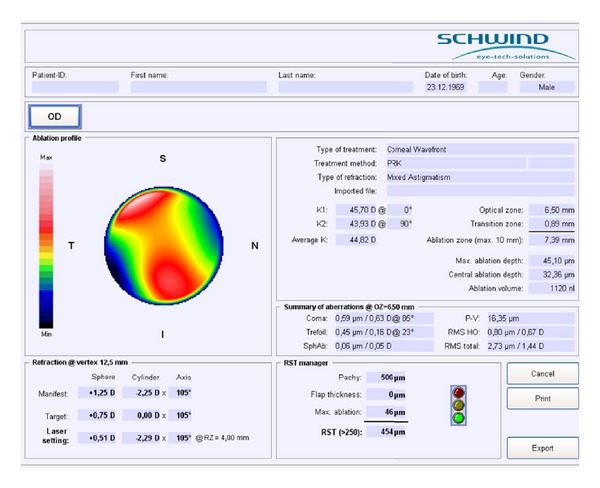
RE ablation map (topographic guided ablation) showing maximum ablation depth of 40.53 *μ*m and central ablation of 30.07 *μ*m.

The patient was then placed on a gurney, his face was covered with a sterile drape, and a blepharostat was inserted. The epithelium was removed with a blunt spatula in the central 8.5 mm optical zone. The patient was submitted to a 6.5 mm optic zone topography-guided PRK in the RE using the Schwind Esiris laser (Schwind eye-tech-solutions GmbH & Co. KG, Kleinostheim, Germany). Maximum ablation depth was 40.53 *μ*m and central ablation was 30.07 *μ*m (Figure 3).

Next, Mitomicina-C 0.02% was applied for 12 seconds in the ablated area and irrigated with 15 mL of balanced saline solution. Following PRK, CXL was performed. A drop of Anestalcon was applied followed by 2 drops of Pilocarpina 2% (Allergan, São Paulo, Brazil). Riboflavin 0.1% was applied with dextran (Ophthalmos, São Paulo, Brazil) every 2 minutes for 15 minutes. The central ultrasonic pachymetry shortly before CXL was 405 *μ*m. UVA light was applied (VEGA CBM X-Linker, CSO, Florence, Italy) in 6 sessions, each lasting 5 minutes, with the application of riboflavin every 3 minutes. Following the procedure, the eye was washed with 20 mL of saline solution 0.9% (Glicolabor, Ribeirão Preto, Brazil). Two drops of gatifloxacin 0.3% together with prednisolone acetate 1% (ZYPRED, Allergan, São Paulo, Brazil) were applied, and a therapeutic contact lens (CL; ACUVUE OASYS, Johnson & Johnson, São José dos Campos, Brazil) was fitted. Finally, an acrylic protector was placed over the operated eye.

Ketorolac tromethamine 0.5% (Acular LS, Allergan, São Paulo) twice daily and Zypred four times a day, starting 2 hours after the procedure, were prescribed. Aceclofenac 100 mg twice daily was prescribed to control postoperative pain, as well as vitamin C 500 mg twice daily for 4 months. In the case of persistent pain, paracetamol with codeine (Tylex, Janssen-Cilag, São Paulo, Brazil) every 6 hours was prescribed.

Since the patient lived 500 km away from the hospital, he was advised to have an ophthalmologist in his town to remove the CL. After removal of the CL, loteprednol 0.5% (Loteprol, Bausch & Lomb, São Paulo) was prescribed three times daily for 1 month and OPTIVE UD (Allergan, São Paulo, Brazil) four times daily for 1 month. 

**Figure 4:**
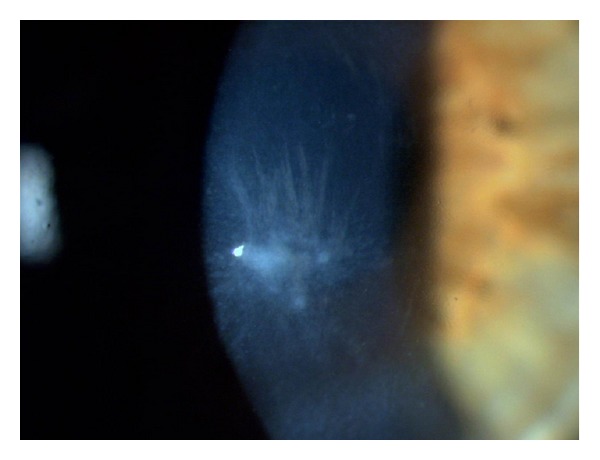
RE biomicroscopy showing hyperplasic epithelial nodule in the central region of the cornea as well as discreet anterior stromal striae.

Forty-five days after surgery, the patient returned to the hospital, complaining of poor vision in the operated eye and claiming no improvement since the surgery. On examination, central epithelial hyperplasia forming a corneal nodule with slight anterior stromal striae was observed (Figure 4).

It was decided to remove the epithelial nodule through simple debridement with a blunt spatula like that used for performing PRK. A CL was fitted (ACUVUE OASYS, Johnson & Johnson, São José dos Campos, Brazil), and ZYPRED drops were prescribed every 4 hours for 1 week and then every 8 hours for another 1 week; OPTIVE (Allergan, São Paulo, Brazil) drops every 4 hours were to be used continuously. The CL was removed after 5 days with normal epithelialization. Discreet stromal striae persisted.

**Figure 5:**
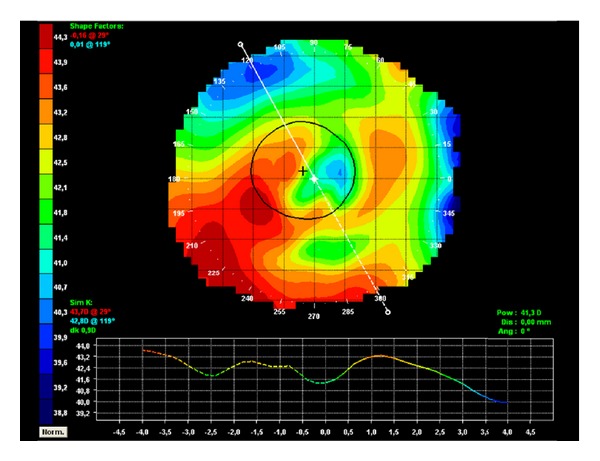
RE corneal topography showing central flattening and irregular astigmatism with stable ectasia after removal of nodule.

Five months after removing the nodule, the patient was still complaining of monocular diplopia. Uncorrected VA was 20/60 and corrected was 20/50, with a dynamic refraction of +2.75, −0.75, 175°. The topography showed central flattening and irregular astigmatism with stable ectasia (Figure 5).

The patient was unable to adapt to rigid gas-permeable contact lenses but attained a VA of 20/25 with soft CL. Weekly followup was opted for.

**Figure 6:**
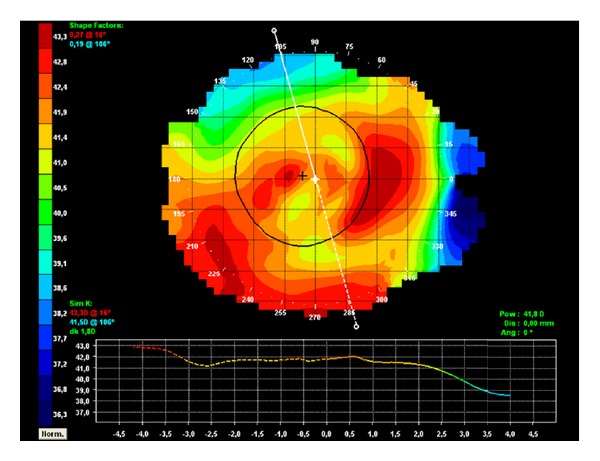
RE topography showing an improvement in the asymmetry 1 year after removal of nodule.

The topography after 1 year showed a significant improvement in central flattening and asymmetry (Figure 6), but the patient continued to experience blurred vision and discreet monocular diplopia. After 2 years, there was no change in the topography in relation to the previous year and the corrected VA was 20/30 with refraction of +2.75, −1.75, 125°. A new topography-guided PRK with static and dynamic cyclotorsion control was opted for. The optic zone was 6.5 mm with a Schwind Amaris laser. The preoperative Orbscan showed 428 *μ*m pachymetry at the thinnest point. After ablation, the stromal residual bed measured by Schwind Amaris laser optic pachymetry, based on low coherence interferometry, was 360 *μ*m.

The patient returned after 4 months with a slight improvement in RE vision. The refraction was −1.50, −1.25, 70° reaching 20/30 VA. Pachymetry was 396 *μ*m. The epithelium showed slight thickening in the center of the cornea with stromal haze of 1+. The topography further showed asymmetry in the visual axis, which should be reduced with epithelial remodeling.

## 3. Discussion

Pairing PRK and CXL techniques has become more frequent, but complications, as yet undescribed in the literature, can occur. To date, there has been no similar case reported in the literature in which a patient was submitted to PRK and CXL consecutively and subsequently developed epithelial hyperplasia forming a central nodule and permanent secondary topographic alteration. 

The PRK technique involves mechanical removal of the epithelium, in addition to photodisruption of the Bowman layer and the anterior stroma by excimer laser, mainly resulting in epithelial hyperplasia and stromal remodeling [6–8]. The first stage of corneal healing after PRK is epithelial migration along the stromal bed [9]. After reepithelialization, compensatory hyperplasia occurs and the basal epithelium anchors to the stroma, forming an adhesion complex [9]. This new epithelium acts as an imperfect barrier, which is permeable to many cytokines and tear film growth factors that can cause cytokine activation [9]. Epithelial hyperplasia, however, occurs as part of the normal corneal healing process. In the case described, hyperplasia was exacerbated, probably due to the inflammatory stimulus triggered by combining PRK with CXL. The formation of linear haze in posterior stroma has been reported in patients submitted to PRK followed by CXL, and the single predisposing factor was the individual response of the patients submitted to the combined treatment [10].

The fact that reepithelialization took longer in some patients who underwent CXL and the broad area of deepithelialization (9 mm) may have contributed to the formation of a hyperplasic nodule, since a delay in epithelialization can lead to epithelial cell hyperplasia on the edge of the lesion [11]. CL contact with the cone apex is another factor that can delay reepithelialization and stimulate hyperplasia.

In this paper, the hyperplasic nodule also caused stromal alteration, with associated striae that generated significant irregular astigmatism in the visual axis. This alteration was probably secondary to the production of collagenolytic enzymes, with consequent stromal collagen degradation. Keratocytes produce type I collagen but can also stimulate the production of type I collagenase [12], which degrades type I, II, and III collagens. The epithelium produces another collagenolytic enzyme, gelatinase, which degrades type IV and V collagens [13]. Furthermore, we know through post-LASIK epithelial ingrowth studies that the epithelial cells that are retained between the flap and the stromal bed present metabolic alterations, which also result in the release of collagenase [13, 14]. The degradation of stromal collagen by these enzymes may have been the cause of the stromal irregularity found in the location where the nodule formed. Furthermore, the possibility of the nodule causing compression and deformation to the adjacent stroma cannot be discarded. The stromal alterations might have been avoided if the hyperplasia had been removed within the first days of its appearance. Thus, it is suggested that patients undergoing CXL should be closely observed by an ophthalmologist familiar with such complications.

We avoided any surgical intervention within the first months after the nodule removal for the purpose of evaluating the final result of the epithelialization process. Since the topography, VA, and refraction were stable after 2 years, we opted for a new ablation based on the topography.
